# A *de novo* microdeletion of *SEMA5A* in a boy with autism spectrum disorder and intellectual disability

**DOI:** 10.1038/ejhg.2015.211

**Published:** 2015-09-23

**Authors:** Anne-Laure Mosca-Boidron, Lucie Gueneau, Guillaume Huguet, Alice Goldenberg, Céline Henry, Nadège Gigot, Emilie Pallesi-Pocachard, Antonio Falace, Laurence Duplomb, Julien Thevenon, Yannis Duffourd, Judith ST-Onge, Pascal Chambon, Jean-Baptiste Rivière, Christel Thauvin-Robinet, Patrick Callier, Nathalie Marle, Muriel Payet, Clemence Ragon, Hany Goubran Botros, Julien Buratti, Sophie Calderari, Guillaume Dumas, Richard Delorme, Nathalie Lagarde, Jean-Michel Pinoit, Antoine Rosier, Alice Masurel-Paulet, Carlos Cardoso, Francine Mugneret, Pascale Saugier-Veber, Dominique Campion, Laurence Faivre, Thomas Bourgeron

**Affiliations:** 1 Laboratoire de Cytogénétique, Plateau technique de Biologie, CHU Dijon, Dijon, France; 2 Equipe Génétique et Anomalies du Développement, Faculté de Médecine, Université de Bourgogne, Dijon, France; 3 Institut Pasteur, Human Genetics and Cognitive Functions Unit, Paris, France; 4 CNRS UMR 3571: Genes, Synapses and Cognition, Institut Pasteur, Paris, France; 5 Université Paris Diderot, Sorbonne Paris Cité, Human Genetics and Cognitive Functions, Paris, France; 6 Centre de Génétique du CHU de Rouen, Rouen, France; 7 Centre Resource Autisme Bourgogne, CHU Dijon, Dijon, France; 8 Institut de Neurobiologie de la Méditerranée INSERM UMR901, Marseille, France; 9 Centre de Génétique et Centre de Référence «Anomalies du Développement et Syndromes Malformatifs», Hôpital d'Enfants, CHU Dijon, Dijon, France; 10 Laboratoire de Génétique Moléculaire, Plateau Technique de Biologie, CHU Dijon, Dijon, France; 11 Fondation FondaMental, Créteil, France; 12 Psychiatrie de l'enfance et de l'adolescence - Hôpital Robert-Debré, Paris, France; 13 Centre de Ressources Autisme de Haute Normandie, Saint Etienne du Rouvray, France; 14 Inserm U 1079, IRIB, Rouen, France

## Abstract

Semaphorins are a large family of secreted and membrane-associated proteins necessary for wiring of the brain. Semaphorin 5A (SEMA5A) acts as a bifunctional guidance cue, exerting both attractive and inhibitory effects on developing axons. Previous studies have suggested that *SEMA5A* could be a susceptibility gene for autism spectrum disorders (ASDs). We first identified a *de novo* translocation t(5;22)(p15.3;q11.21) in a patient with ASD and intellectual disability (ID). At the translocation breakpoint on chromosome 5, we observed a 861-kb deletion encompassing the end of the *SEMA5A* gene. We delineated the breakpoint by NGS and observed that no gene was disrupted on chromosome 22. We then used Sanger sequencing to search for deleterious variants affecting *SEMA5A* in 142 patients with ASD. We also identified two independent heterozygous variants located in a conserved functional domain of the protein. Both variants were maternally inherited and predicted as deleterious. Our genetic screens identified the first case of a *de novo SEMA5A* microdeletion in a patient with ASD and ID. Although our study alone cannot formally associate *SEMA5A* with susceptibility to ASD, it provides additional evidence that Semaphorin dysfunction could lead to ASD and ID. Further studies on Semaphorins are warranted to better understand the role of this family of genes in susceptibility to neurodevelopmental disorders.

## Introduction

Autism spectrum disorders (ASDs) are characterized by impairments in social interactions, stereotypy and a restricted repertoire of activity and interest. ASDs affect 0.6–1.5% of the population, with 4–8 times more males diagnosed than females.^1, 2^ ASDs are etiologically heterogeneous and are associated with an identified genetic etiology in about 20% of cases.^3, 4, 5^ ASD can be associated with known genetic disorders such as fragile X syndrome, tuberous sclerosis (*TSC1*, *TSC2*), neurofibromatosis (*NF1*), Angelman syndrome (*UBE3A*), Rett syndrome (*MECP2*), and Cowden syndrome (*PTEN)*. Microscopically visible chromosomal alterations and copy-number variations (CNVs) account for 3–10% of cases.^6^ Finally, *de novo* disruptive variants are more frequent in patients with ASD than in their unaffected siblings and controls.^7, 8, 9, 10, 11^ The role of inherited variants remains unclear, but it was estimated that at least 6% of cases could be caused by rare recessive variants affecting both alleles and that common variants act in an additive manner to increase the genetic risk to autism.^12, 13, 14^ Based on current findings, the genetic architecture of ASD is highly heterogeneous, with many genes/loci and possibly gene–gene interactions involved.^4, 5^

Genes associated with ASD participate in at least three main biological processes.^4, 5, 15^ The first pathway is related to chromatin remodeling and includes genes such as *MECP2* (mutated in patients with Rett syndrome) and *CHD8*. The second pathway is related to the PI3K-mTOR signaling pathway and includes variants in *NF1*, *TSC1*, *TSC2*, and *PTEN* that normally inhibit translation.^16^ Downstream of the PI3K-mTOR signaling pathway, proteins directly involved in the inhibition of mRNA translation at the synapse (FMR1, CYFIP1, EIF4E) are also mutated in ASD.^17^ The third pathway concerns the balance between excitation and inhibition and the formation and maintenance of synapses.^5, 18^ At the synapse, cell adhesion proteins such as neuroligins and neurexins and scaffold proteins such as SHANK3 are involved in dendrite formation and the assembly of synapses.^19^ In addition, epilepsy-associated voltage-gated sodium channels such as *SCN1A* were also found mutated in patients with ASD. Finally, guidance cues for axonal outgrowth such as semaphorins were also associated with ASD suggesting that abnormal wiring of the brain could be a risk factor for ASD.^18^ The semaphorin *SEMA5A* gene was associated with ASD according to independent results from (i) genome-wide association studies (GWAS)^20, 21^ and expression assay,^22^ (ii) RNA profiling of brains^20^ and B lymphoblastoid cell lines^22^ from patients with ASD, and (iii) pathway analysis using expression quantitative traits loci (eQTL) and CNV data.^23^ SEMA5A is also one of the genes deleted in the minimal region in patients presenting with ‘cri-du chat' syndrome and a subset of these patients present autistic traits including repetitive movements, obsessive attachment to objects, hypersensitivity to sensory stimuli, gaze avoidance, and social isolation.^24^

**Figure 1:**
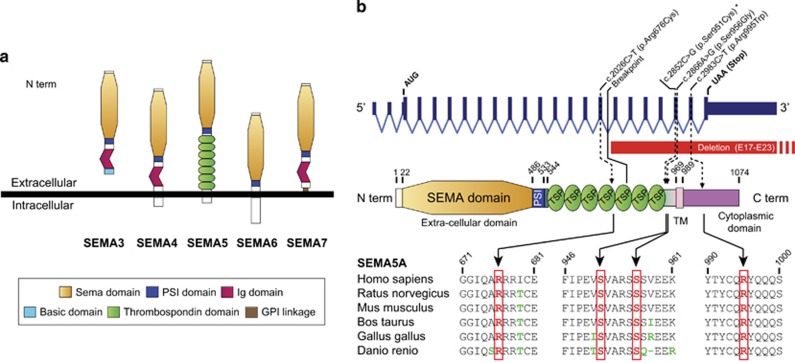
Semaphorin phylogeny and structure. (**a**) Primary structures of the semaphorin family in mammals. (**b**) Structure of the SEMA5A protein and locations of the coding variants (c.2026C>T, c.2852C>G, c.2866A>G, c.2983C>T). The microdeletion identified in this study is indicated in red and the star represents the *de novo* missense variant (c.2852C>G) reported by Lossifov *et al.*^35^ The conservation of the amino acids is indicated for different species. TM, transmembrane domain; TSP, thrombospondin repeat.

Semaphorins are a large and diverse family of secreted and membrane-associated proteins, which are conserved both structurally and functionally across divergent animal phyla^25, 26^ (Figure 1a). All members of this family contain a conserved extracellular domain of about 500 amino acids, termed the semaphorin domain, which is characterized by highly conserved cysteine residues that have been found to form intrasubunit disulfide bonds (Figure 1b). Class 5 semaphorins (SEMA5A and SEMA5B) are characterized by the presence of seven type 1 thrombospondin repeats in their extracellular domain. Both SEMA5A and SEMA5B seem to play a crucial role in the development of neuronal circuits.^27, 28, 29, 30^ Using a rat model, Kantor *et al.*^29^ found that SEMA5A acts as a bifunctional guidance cue, exerting both attractive and inhibitory effects on developing axons of the fasciculus retroflexus, a diencephalon fiber tract associated with limbic function. More recently, Duan *et al.*^28^ showed that SEMA5A negatively regulates synaptogenesis in early, developmentally born, hippocampus. SEMA5A is strongly expressed by the hippocampal dentate granule cells and regulates dendritic spine density in a cell-autonomous manner.^28^ Mice lacking SEMA5A display deficits in social interaction, a hallmark of ASD.^28^

Here, we report the first case of a *de novo* translocation t(5;22)(p15.3;q11.21) associated with a partial deletion of *SEMA5A* in a patient with ASD. In order to determine the contribution of *SEMA5A* to ASD, we then ascertained the clinical characterization of this patient and screened for *SEMA5A* variants.

## MATERIALS AND METHODS

### Cohorts of patients with ASD

To identify SEMA5A coding variants, we sequenced a cohort of 142 patients (121 males) from three different French genetic centers (Paris, Rouen, and Dijon). Affected individuals were diagnosed using the Autism Diagnostic Interview-Revised (ADI-R)^31^ and/or the Autism Diagnostic Observation Schedule (ADOS) according to DSM-IV and ICD-10 criteria. Standard karyotype, fragile X testing, and metabolic screening for inherited metabolism disorders (plasma and urinary amino acids, urinary mucopolysaccharides and organic acids, urinary purines and pyrimidines, urinary creatinine, and guanidoacetate) showed no abnormalities. There were no specific inclusion criteria for the screening for *SEMA5A* coding variants. The cohort of 142 patients consisted of 87% of individuals with autism, 10% of individuals with atypical autism (including PDD-NOS), and 3% of individuals with Asperger syndrome. The cohort of CNVs consisted of 996 cases and 1287 controls from the Autism Genome Project, and 296 patients and 509 controls from our laboratory. In accordance with ethical guidelines, written informed consent was collected from all patients or from their parents or guardians, and from all the other participating careers before inclusion in the study. The approval of the ethics committee of Dijon University Hospital was not required since the analyses were performed as part of the diagnostic work-up and did not require additional samples. The parents or guardians of the patients signed an informed consent form for publication of the data.

### Standard and molecular cytogenetic studies

After clinical examination by a geneticist, the chromosomes of the proband and his parents were obtained from peripheral blood lymphocyte cultures using standard cytogenetic analysis with R and G banding techniques. Whole-chromosome painting analysis of chromosomes 5 and 22 was performed in the proband. CNVs were detected using the Human Genome Microarray CGH 44 K, from Agilent according to the manufacturer's protocol (Agilent Technologies, Santa Clara, CA, USA) and different Illumina SNP arrays (Human Omni 1, Omni 2.5, and Omni 5 BeadChip arrays). CGH data were processed with Feature Extraction (v. 9.1) software and the results were analyzed with CGH analytics (v. 4.0) software (Agilent). Mapping data were analyzed on the human genome sequence using Ensembl (www.ensembl.org; Hg 19). CNVs were assessed in the Database of Genomic Variants (http://projects.tcag.ca/variation/). CNV identified by microarray analyses was confirmed by fluorescence *in situ* hybridization (FISH) using BACs RP11-747E07 and RP11-57N07 (The Human 32 K clone set, CHORI) on metaphase chromosome preparations and by quantitative PCR targeting the *SEMA5A* gene in the proband and in his parents. Real-time PCR was performed in the LightCycler 480 system (Roche) using the SYBR Green I Master Kit (Eurogentec, Seraing, Belgium) with 2  μl of cDNA and 200 nm of each primer. Each reaction was performed in triplicate.

### Sequencing of SEMA5A

Genomic DNA was extracted from EDTA-blood by the salting-out method. PCR amplification of all 21 *SEMA5A* coding exons and exon–intron boundaries was performed using the Taq DNA polymerase from Invitrogen. All PCR products were directly sequenced using an ABI Genetic Analyser 3100 capillary sequencer according to the manufacturer's instructions (Applied Biosystems, Foster City, CA, USA). The reference sequence of *SEMA5A* genomic DNA was downloaded using Ensembl Genome Browser (accession number ENSG00000112902). Nomenclature for the description of sequence variants was based on the current Ensembl transcript (Ensembl Transcript ID ENST00000382496) with the position +1 as the A of the ATG initiation codon. Collected data were analyzed with SeqScape v2.7 software (Applied Biosystems).

### Next-generation sequencing of the translocation breakpoints

Breakpoint detection was based on whole-genome sequencing with the paired-end protocol and specific bioinformatics analysis (Integragen, Evry, France). Library preparation, sequencing, and variant detection and annotation were performed by IntegraGen. The generated paired-end libraries were prepared according to the Agilent Technologies process. For detailed explanations of this process, please see SureSelect XT2 Target Enrichment System for Illumina Multiplexed Sequencing Protocol (version B, April 2012). Note that only the pre-capture step was processed to generate paired-end libraries and the shearing process was modified to yield long DNA fragments. Briefly, 1 μg of genomic DNA was fragmented by sonication and purified. The mode of the resulting fragment-size distribution was approximately 400 bp. Indexed paired-end adaptor oligonucleotides were ligated on repaired A-tailed fragments, then purified and enriched by four PCR cycles to increase the yield. Each DNA library was quantified by qPCR using specific Illumina oligonucleotides and then sequenced on an Illumina HiSeq 2000, where genomic paired-end reads of 2 × 100 nucleotides were generated. Image analysis and base calling were performed using Illumina Real Time Analysis Pipeline version 1.14 with default parameters.

The bioinformatics analysis of sequencing data was based on BWA for the alignment step, and on *GASV* software for breakpoint detection. Reads alignment was performed with multiseed and gapped alignments on reference human genome hg19 and reads were paired with a median fragment size of 300 bp. Sequences with more than two mismatches were excluded, as were duplicated sequences corresponding to PCR amplification bias. Then, from the alignment, a list of reads mapped to different chromosomes for translocations was retained. Finally, only abnormalities supported by three independent pairs of reads were verified. If this analysis scheme was not sufficient to identify the breakpoints, six mismatches per sequence were tolerated.

## Results

We analyzed the contribution of *SEMA5A* variants in ASD using a panel of genetic technologies including standard karyotyping and SNP arrays, as well as Sanger and next-generation sequencing. We report for the first time on a 4-year-old-boy with ASD carrying a *de novo* translocation leading to a partial deletion of *SEMA5A* on chromosome 5. He was referred to the Genetics Department because of psychomotor delay associated with abnormal behavior. The pregnancy was uneventful and he was born at 36 weeks and 5 days of gestation by caesarean section because of a breech presentation. The birth measurements were as follows: weight: 2.4 kg, length: 43 cm, and occipitofrontal circumference (OFC): 33 cm. He had suffered from asthma since the first month of his life. He was the third of three siblings and his two brothers had speech delay that was rapidly corrected and did not require any further investigation. At 4 years of age the physical examination revealed normal growth parameters (weight: 17 kg, height: 100 cm, and OFC 51.5 cm) and there were no discernible dysmorphic features. In particular, he presented no features of ‘cri-du chat' syndrome such as microcephaly, hypertelorism, and hypotonia. He had no visceral malformation, a normal cardiac, and renal ultrasound. Brain MRI was reported as normal. His reciprocal interactions and communication skills were impaired. He showed evidence of the first symptoms of ASD at 18 months of age as he did not react to noise (hearing was tested and appeared normal), and at 2 years he presented with speech delay. At the age of 4 years he could only pronounce sounds. The history of his early development was difficult to assemble. The diagnosis of childhood autism was ruled out according to the Autism Diagnosis Interview-Revised because of the absence of restricted and stereotyped patterns of interest.^31^ He met the ICD-10 criteria for PDD—not otherwise specified as he had severely impaired verbal and non-verbal communication, and impaired social interactions. Moreover, at 5 years of age, the results of the Psychoeducational Profile-Revised (PEP-R) indicated a developmental score equivalent to an age of 17 months and confirmed the associated diagnosis of intellectual disability (ID).

**Figure 2:**
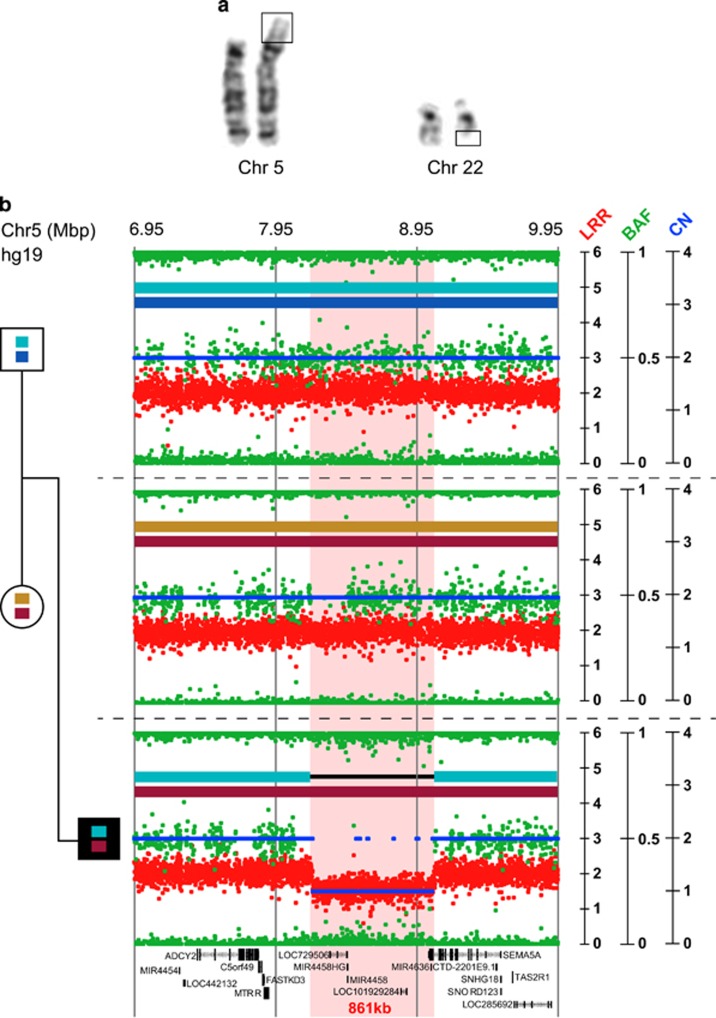
Characterization of the chromosomal rearrangement leading to the microdeletion of the *SEMA5A* gene. (**a**) Partial G-banded karyotype showing the translocation t(5;22)(p15.3;q11.21) of the proband. (**b**) Results of the SNP-array (Illumina Human Omni 2.5) analysis showing the *de novo* 861-kb deletion (chr5:8205612-9068974 hg19) of the region 5p15.3 including the seven last exons of *SEMA5A*. Based on informative SNPs located within the deletion, we ascertained that the deletion was on the father's chromosome. Each dot shows log *R* ratio (LRR; in red), the B allele frequency (BAF; in green), and the copy number (CN; in blue).

The karyotype analysis of the patient and his parents revealed a *de novo* translocation 46,XY, t(5;22)(p15.3;q11.2) (Figure 2a). Whole-chromosome painting analysis of chromosomes 5 and 22 showed that the chromosomal rearrangement involved only these two chromosomes (data not shown). To determine whether there were any losses or gains of material around the breakpoints of the translocation, we performed SNP/CNV analysis using the Illumina 2.5 M array (Figure 2b). At the translocation breakpoint on chromosome 5, we observed an 861-kb deletion in the 5p15.3 region (arr[hg19] 5p15.3(8,205612–9,066,802) × 1 dn). The deletion was confirmed by FISH and qPCR and was *de novo* since it was only observed in the proband and not in the parents. Next-generation sequencing defined the breakpoint in a 361-bp region on chromosome 5 at 5p15.3 (chr5:g.90685589_9068950, hg19) and on chromosome 22 in a repetitive element at 22q11.21 (chr22:g.18718082_18718443, hg19). No gene was disrupted on chromosome 22. We therefore confirmed that the patient was carrying a *de novo* partial deletion of the *SEMA5A* gene (exons 17–23). The deletion has been submitted to Decipher (patient ID 280507, URL: https://decipher.sanger.ac.uk/). The Database of Genomic Variants reported only one intragenic deletion (nsv597070).

In order to detect additional *SEMA5A* variants in patients with ASD, we sequenced all coding exons of the gene in 142 independent patients. We found one nonsynonymous variant (c.2866A>G p.(Ser956Gly) NM_003966.2) already listed in dbSNP and two nonsynonymous variants (c.2026C>T p.(Arg676Cys) and c.2983C>T p.(Arg995Trp) NM_003966.2). The first one has never been reported in the ExAC and in the Exome Variant Server (EVS) databases, but a neighboring variant c.2027G>A p.(Arg676His) has been reported in the ExAC database at a very low frequency (MAF=0.00001666). The second one was present in the ExAC database in a similar very low frequency (MAF=0.00001666). Both variants c.2026C>T and c.2983C>T were located in highly conserved regions of the thrombospondin and the transmembrane domains, respectively, and have been submitted to LOVD (patients IDs 00037526 and 00037568), URL: www.lovd.nl/SEMA5A (Figure 1b). These variants were maternally inherited and predicted as deleterious by Polyphen2 and SIFT algorithms. Based on the EVS, rare *SEMA5A* variants are found in 0.56% of individuals from European ancestors (ASD: 2/142 vs EVS controls 24/4300; Fisher's exact test, two-tailed: *P*=0.2). Based on the recent screen for *de novo* variants in ASD, a *de novo* missense variant of *SEMA5A* (c.2852C>G p.(Ser951Cys)) affecting a conserved amino acid and predicted as deleterious was identified in a female with ASD.

In our study, the patient carrying the c.2026C>T variant is a 6-year-old boy, the second child of non-consanguineous parents. The family history revealed a schizophrenic paternal uncle. The pregnancy was normal and he was born at 37 weeks of gestation. Birth weight was 2870 g (3rd centile) and OFC was at the 95th centile (36.5 cm). He showed evidence of the first symptoms of ASD at 20 months of age as he had psychomotor delay and abnormal behavior. He had no visceral malformation, in particular a normal cardiac and renal ultrasound. Brain MRI was reported as normal. He walked between 16 and 18 months of age and said his first words between 8 and 12 months of age. At the age of 1 year he stopped talking and showed impaired social reciprocity, slightly aggressive behavior, and stereotyped hand movements. He was referred to a psychiatric unit at 4 years of age because of deficits in verbal communication associated with repetitive behaviors and was diagnosed with autism, according to DSM-IV and ICD-10 criteria. The severity of the autistic behavior was assessed using the Childhood Autism Rating Scale scores. The score of 41.5 corresponded to moderate to severe ASD and, despite his psychomotor delay, he did not present ID. When examined at 6 years of age, he was 116 cm tall (50th centile), weighed 21 kg (50th centile), and had an OFC of 56 cm (>97th centile); the neurological examination was normal. Presence of a variant of the PTEN gene and other pathogenic CNVs was excluded by Sanger sequencing and 105 K CGH-array, respectively. He presented mild dysmorphism including hypertelorism and a wide mouth. He used non-verbal communication and had aggressive behavior.

The patient carrying the maternally inherited c.2983C>T variant is a 6-year-old boy, the second child of non-consanguineous parents. The family history was uneventful, except for the mother who experienced two episodes of depression, one at 11 years of age and the second at age 36. Her psychiatric evaluation showed good reciprocal social interactions. The pregnancy was marked by maternal hepatic steatosis and the proband was born at 37.5 weeks of gestation. Birth weight was 2760 g (3rd centile), length 49 cm (50th centile), and an OFC of 31.5 cm (<3rd centile). Apgar scores were 4 and 9 at 1 and 5 min, respectively. At 1 month of age he presented with hypotonia, motor agitation, and no babbling. He walked at 18 months and he could only say two words at 2 years. He had no visceral malformation, in particular a normal cardiac and renal ultrasound. Brain MRI was reported as normal. He was referred to a psychiatric unit because of aggressive behavior and speech delay. He was diagnosed with ASD, according to DSM-IV and ICD-10 criteria. He met the criteria for autism according to ADI-R and ADOS. His cognitive level was in the normal range. When examined at 6 years of age he was 122 cm tall (97th centile), weighed 25 kg (97th centile), and had an OFC of 50 cm (50–25th centile). The neurological examination was normal and no pathogenic CNVs were detected by the 105 K array-CGH.

## Discussion

In the literature, multiple lines of evidence support a role for *SEMA5A* in susceptibility to ASD. First, a large-scale GWAS performed on 1031 independent families with ASD initially found a statistically genome-wide significant association between ASD and rs10513025 located 5′ to *SEMA5A*.^20^ Following this GWAS, an association between the same SNP and ASD was also detected using the transmission disequilibrium test in 227 Italian families with ASD.^21^ Another common SNP of *SEMA5A* (rs42352) was associated with hippocampal volume and memory performance in 329 healthy Chinese adults.^32^ Second, in a recent GWAS follow-up approach analyzing SNPs that affect gene expression (eQTL), Cheng *et al.*^23^ found that the SEMA5A regulatory network significantly overlaps with rare autism-specific CNVs. The SEMA5A regulatory network includes previous ASD candidate genes and regions including *MACROD2*, *CDH8*, *FOXP1*, *AUTS2*, *MBD5*, among others.^23^ Third, two independent studies have reported a reduced level of *SEMA5A* mRNA in Epstein–Barr virus-transformed B lymphocytes^22^ or in the brains of patients with ASD.^20^ Finally, *SEMA5A* is located on chromosome 5 in a minimal region deleted in patients with ‘cri-du chat' syndrome. A subset of these patients presents with autistic traits including repetitive movements, obsessive attachment to objects, hypersensitivity to sensory stimuli, gaze avoidance, and social isolation.^24^

While these studies suggest that SEMA5A plays a role in susceptibility to ASD, none of them could formally implicate SEMA5A. In this study, we report the first microdeletion of *SEMA5A* caused by an unbalanced reciprocal translocation in a patient with ASD and severe speech delay. The deletion (exons 17–23) found in the boy with ASD caused the loss of seven exons coding the last five thrombospondin repeats, as well as the transmembrane and the intra-cytoplasmic domains. SEMA5A CNVs are very rare events since they were not detected in the large cohort of ASD patients screened by Pinto *et al.*^6^ Our mutation screening also identified two patients with ASD carrying private missense variants located in highly conserved regions of SEMA5A and predicted to alter protein function. Remarkably, the c.2026C>T p.(Arg676Cys) variant alters an arginine residue that is highly conserved in many thrombospondin domains outside the semaphorin family suggesting that this amino acid is important for correct protein folding. The thrombospondin repeats of SEMA5A were shown to be crucial to promote axon outgrowth through the interaction with proteins such as PlexinA2, but the exact mapping of the protein–protein interaction remains unknown.^28, 29, 33^

To conclude, our study reports the first *de novo* microdeletion of *SEMA5A* in humans. The phenotype of the patient might be consistent with a role of *SEMA5A* in patients with ASD and ID without dysmorphic features. Together with the recent results from the whole-exome sequencing in ASD, this variation is the second *de novo SEMA5A* variation reported in ASD.^34, 35^ As for other inherited rare variants identified in complex disorders, we cannot formally prove that the two missense variants identified in this study represent risk factors for ASD. These variants affected highly conserved amino acids, but were inherited from asymptomatic mothers, thus signifying that if they do play a role in ASD, they might be only one of the factors contributing to the disorder. Indeed, a combination of rare inherited variants was reported in a subset of patients, suggesting a ‘multiple hit' model of ASD.^36, 37^ Altogether, the role of *SEMA5A* variants in ASD seems limited and very large-scale studies are warranted to associate this gene to ASD, but given the importance of semaphorins in the wiring of the brain, any information on the clinical consequences of deleterious variants affecting this family of proteins remains very helpful to understand the numerous neurobiological mechanisms leading to neurodevelopmental disorders.
